# Supplementary material for: Labor prior to cesarean delivery associated with higher post-discharge opioid consumption
Source: PLoS One. 2021 Jul 9;16(7):e0253990. doi: 10.1371/journal.pone.0253990 (PMC8270408; doi:10.1371/journal.pone.0253990)
Supplement: Attachment — Submitted filename: Labor CD Pain PLOS ONE Response to Reviewers 5.13.21.docx [file pone.0253990.s001.docx]

**General Comments:**

Comment 1: Please ensure that your manuscript meets PLOS ONE's style requirements, including those for file naming. The PLOS ONE style templates can be found at

<https://journals.plos.org/plosone/s/file?id=wjVg/PLOSOne_formatting_sample_main_body.pdf> and

[https://journals.plos.org/plosone/s/file?id=ba62/PLOSOne_formatting_sample_title_authors_affiliations.pdf](about:blank)

*Response 1: We confirm that we have reviewed the PLOS ONE style requirements to ensure this manuscript meets those criteria.*

Comment 2: Please provide additional details regarding participant consent. In the ethics statement in the Methods and online submission information, please describe how verbal consent was documented and witnessed, and why written consent was not obtained.

*Response 2: This additional detail has been added to the Methods section, under the subheading of Subject enrollment and consent – “…verbal consent was obtained from all subjects by the primary investigator or a co-investigator prior to proceeding with the interview. Written consent was not required by the IRB at these centers. Participant consent was documented in the REDCap (Research Electronic Data Capture) database used for data collection.”*

Comment 3: In your Methods section, please provide additional information about the participant recruitment method and the demographic details of your participants. Please ensure you have provided sufficient details to replicate the analyses such as:
a) a description of any inclusion/exclusion criteria that were applied to participant recruitment
b) a statement as to whether your sample can be considered representative of a larger population

*Response 3: These additional details have been added to the Methods section, under the subheading of Subject enrollment and consent – “Participants represented a convenience sample of women who had undergone either elective or unplanned cesarean delivery at each center and were subsequently identified by the study team by daily rounding, review of the electronic medical record, or notification by the obstetric team. All women post-cesarean delivery were considered for inclusion unless one of the following exclusion criteria were met: limited English proficiency, lack of capacity to provide consent, age <18 years, or hospital length of stay >7 days following delivery.” A statement on generalizability and representativeness of our sample is provided in the Discussion section – “the patients included in the original survey study represented a convenience sample of women undergoing CD at six academic medical centers across the United States, which may not be representative of other practice settings.”*

Comment 4: Please include a copy of the structured interview guide used in the study, in both the original language and English as Supporting Information, or include a citation if it has been published previously.

*Response 4: The hyperlink to the structured interview guide has added to the Methods section, under the subheading of Data Collection.*

Comment 5: We noted in your submission details that a portion of your manuscript may have been presented or published elsewhere. "This is a secondary analysis of a prospective cohort study evaluating patterns of opioid use after cesarean delivery (Bateman BT, et al. Patterns of Opioid Prescription and Use After Cesarean Delivery. Obstet Gynecol. 2017;130(1):29-35). Original publication is attached as Related Manuscript File." Please clarify whether this publication was peer-reviewed and formally published. If this work was previously peer-reviewed and published, in the cover letter please provide the reason that this work does not constitute dual publication and should be included in the current manuscript.

*Response 5: The original publication “Patterns of Opioid Prescription and Use After Cesarean Delivery” was peer-reviewed and published in Obstetrics & Gynecology in 2017. The current study is a secondary analysis of the data collected for that previous study, but represents a completely novel hypothesis, data analysis, and reporting of results. No outcomes or results from the prior study are re-presented in the current work. This detail has been added to the updated Cover Letter.*

Comment 6a: If there are ethical or legal restrictions on sharing a de-identified data set, please explain them in detail (e.g., data contain potentially identifying or sensitive patient information) and who has imposed them (e.g., an ethics committee). Please also provide contact information for a data access committee, ethics committee, or other institutional body to which data requests may be sent. If there are no restrictions, please upload the minimal anonymized data set necessary to replicate your study findings as either Supporting Information files or to a stable, public repository and provide us with the relevant URLs, DOIs, or accession numbers.

*Response 6a: This data represents sensitive patient information involving self-reported patient opioid use and pain scores. As we did not request permission for release of the de-identified dataset in the Institutional Review Board applications at all of the institutions included in the study, it is not ethically appropriate to release this sensitive information, even in deidentified form. Furthermore, while restrictions are not specifically imposed by any organization, public release of the data was not discussed with patients during the consent process and thus we cannot release patient-level data.*

*Requests for data can be forwarded to:*

*Massachusetts General Hospital Anesthesia Research Center*

*c/o: Ariel Mueller*

*55 Fruit Street, White 5*

*Boston, Massachusetts 02114*

*Email:* [*almueller@mgh.harvard.edu*](mailto:almueller@mgh.harvard.edu)

*Phone: 617-726-9252*

Comment 7: During our internal evaluation of the manuscript, we found that your abstract appears to have been previously published with the Society for Obstetric Anesthesia and Perinatology. Before we proceed, would you please kindly clarify if the published extended abstract was previously peer-reviewed? If so, please explain in your cover letter why this work does not constitute a dual publication. Please also clarify at this time whether your abstract was previously copyrighted.

*Response 7: The current work was presented in abstract form at the Society for Obstetric Anesthesia and Perinatology Annual Meeting in 2017. The abstract was not peer-reviewed or published. It was included only as part of the Annual Meeting Syllabus. Neither the abstract nor the current manuscript are copyrighted in any way.*

**Reviewer 1:**
Comment 1: Thank you for your work. The results are consistent with the research hypothesis. The discussion covers the analysis of the results with comparison with other articles. tables are clear. I recommend publishing the paper

*Response 1: Thank you for taking the time to review our manuscript and for this kind review of our paper.*

**Reviewer 2:**

Comment 1: ABSTRACT: There are just few grammatical errors in the abstract that need to be corrected.

*Response 1: Thank you for bringing our attention to any grammatical errors. We have edited punctuation on lines 61 and 78 of the Abstract.*

Comment 2: METHODS: Why were these 6 centers chosen for the study and why did some of the participants give a written consent and a verbal consent obtained from the others (page 5 lines 114 and 116)

*Response 2: Additional detail has been added to the Methods section regarding consent procedures at the varying institutions. The six centers were chosen to represent a sampling of tertiary academic medical centers throughout the United States, in varying geographic regions.*

Comment 3: Apart from that this secondary analysis of a previous prospective cohort study is acceptable for publication.

*Response 3: Thank you for taking the time to review our manuscript and for your positive reception of our work.*

**Additional Requests from Editorial Office Email dated 5/7/21**

Comment 1: Please indicate the policy, ethics committee, Institutional Review Board, or other organization that is imposing the restriction.

*Response 1: While restrictions are not specifically imposed by any organization, public release of the data was not discussed with patients during the consent process and thus we cannot publicly release patient-level data.*

Comment 2: Provide a non-author, institutional point of contact (and contact information) that is able to field data access queries. PLOS ONE's data policy requires this in the interest of maintaining long-term data accessibility.

*Response 2: Requests for data can be forwarded to:*

*Massachusetts General Hospital Anesthesia Research Center*

*c/o: Ariel Mueller*

*55 Fruit Street, White 5*

*Boston, Massachusetts 02114*

*Email:* [*almueller@mgh.harvard.edu*](mailto:almueller@mgh.harvard.edu)

*Phone: 617-726-9252*

Comment 3: If relevant, add any data set names, variables, accession codes, URLs, DOIs, etc. that an independent researcher would need in order to request your minimal data set.

*Response 3: Not applicable. Please see responses to comments 1 and 2 above.*

Comment 4: Thank you for providing the following response to our previous dual publication query: "The original publication “Patterns of Opioid Prescription and Use After Cesarean Delivery” was peer-reviewed and published in Obstetrics & Gynecology in 2017. The current study is a secondary analysis of the data collected for that previous study, but represents a completely novel hypothesis, data analysis, and reporting of results. No outcomes or results from the prior study are re-presented in the current work. This detail has been added to the updated Cover Letter." Before we proceed, would you please kindly clarify the following points:

Did the authors present any new data in this submission that were not previously presented in the published article Bateman BT, et al.? Did the authors perform any additional experiments or collect any additional data that were not a part of the study from the published article Bateman BT, et al.?

*Response 4: The current work represents a new analysis of the data which were previously collected for the study published by Bateman BT, et al. For the previously published study, women were surveyed and reported their pain scores and number of opioid tablets consumed for two weeks following cesarean delivery. The study reported the median number of dispensed opioid tablets and the number of leftover tablets at the time of interview. In the current work, we tested the hypothesis that the number of opioid tablets consumed post-cesarean delivery would be greater in those patients who underwent intrapartum, as opposed to scheduled, cesarean delivery. This represents a new hypothesis, new data analysis, and reporting of new results not previously published in the 2017 article by Bateman BT, et al.*

Comment 5: If the authors are conducting a "secondary analysis of the data collected for that previous study", please clarify whether the authors have the rights to both reuse and republish this data under a CC BY 4.0 license.

*Response 5: We confirm that the data collected is owned by the investigators and that the authors have the rights to both reuse and republish the data under a CC BY 4.0 license.*
