# Supplementary material for: Labor prior to cesarean delivery associated with higher post-discharge opioid consumption
Source: PLoS One. 2021 Jul 9;16(7):e0253990. doi: 10.1371/journal.pone.0253990 (PMC8270408; doi:10.1371/journal.pone.0253990)
Supplement: Attachment — Submitted filename: Response to Reviewers 5.26.21.docx [file pone.0253990.s002.docx]

***Response to Reviewers***

***PONE-D-20-40071R1
Labor prior to cesarean delivery associated with higher post-discharge opioid consumption***

We again would like to thank the handling editor and reviewers for taking the time to review our revised manuscript and make additional suggestions on how it can be further improved. Below we outline all changes made during this second revision and have marked all changes appropriately in the corresponding manuscript files. We hope that with these additional amendments you will find our work suitable for publication in *PLOS One*.

**Changes to Reference List**

Journal abbreviations have been corrected for references numbers 8 and 12.

**Reviewer 3**
Comment 1: The study was well-structured. The paper is well written. The authors faithfully reacted to the reviewer’s and editor’s comments. The paper has become markedly improved. I have only one concern: I believe that in this study unplanned intrapartum CS vs. planned before-labor CS has been compared, right? All the context tells me so. Let us sort out the terminology of cesarean section from two viewpoints: planned vs. unplanned and labor + vs. -. Naturally, there are four categories. 1) planned labor (+): breech presentation planned for CS tomorrow but night before labor occurred. Waiting three hours (she had pains every 10 minutes at CS), she had cesarean section at the planned data/time. This may be an extreme but we sometimes encounter the case in which “just before planned cesarean section, we found every ten minutes uterine contractions”. This apparently is defined as after labor (labor initiation is defined as ever ten minutes contraction with progressing cervical findings/ripening; the latter cannot be confirmed due to having done CS, thus every ten minutes contraction is sufficient to define “labor +”). 2) planned labor (-): no need to explain. 3) unplanned (or emergent) labor (+): failure to progress. 4) unplanned labor -; cardiotocography revealed non-reassuring fetal status requiring emergent CS. The context strongly suggest that you here compared 2) vs. 3).

*Response 1: Here we are comparing intrapartum CD (#1 and #3) versus CD without prior labor (#2 and #4). In order to improve transparency, we have adjusted the wording throughout the manuscript in various locations, as you’ve suggested below. The presence or absence of labor was determined by chart review by a study coinvestigator, so we cannot exclude the possibility of rare instances where infrequent contractions prior to a scheduled delivery were not captured in our study (#1 above).*

Comment 2: Here, you used many terminologies, planned, unplanned, elective, non-elective, emergent, scheduled, unscheduled, labor (+), no-labor, intrapartum, etc. As an experienced obstetrician (4.5-decade of practice), I understand the situation; however, what is labor? What is planned? What is emergent? Please definitely state their criteria. For example, labor +/- might depend only presence/absence of every-ten-minute-contraction, right? As I described, one cannot confirm the cervical change and whether “this contraction” led to eventual delivery (not false pains) because some patients underwent CS. Thus, one may use the terminology “labor” under incomplete definition/criteria. Thus, please definitely define these terminologies and state what vs. what was actually compared here. I am not an English-native and thus I feel how wonderful it would be if you had used “consistent terminology”; planned CS vs. scheduled CS is “identical”, right? If so, please use the consistent terminology for the readers. However, if for USA (English native) people/doctors, the present terminology (variety of words) may be better, you can “ignore” my comment/advice. Sorry to write long.

*Response 2: We have adjusted the wording in the manuscript to use consistent terminology (intrapartum vs. unlabored). All mentions of planned, unplanned, elective, non-elective, emergent, scheduled, and unscheduled have been removed from the manuscript (unless they describe the results of other studies).*

Comment 3: For your convenience, I write parts that might cause confusion (due to terminology). From this viewpoint, there are many parts with mixed terminology also in discussion section. Do previous article (data) adopt the same criteria of labor + vs. -? Please confirm it. You need not write long.

*Response 3: See responses to below comments. The terminology used to describe the results of prior articles is consistent with the methods and results described by those studies (ex. planned vs. unplanned or emergent vs. elective is used when that is how they were described in prior studies).*

Comment 4: Introduction last: We hypothesized that intrapartum CD, which has been associated with both increased inflammation[11] and affective distress related to an unexpected CD,[12] would result in higher postoperative pain scores and increased opioid intake compared to scheduled CD without prior labor.

*Response 4: The wording has been adjusted for consistency – “We hypothesized that intrapartum CD, which has been associated with both increased inflammation[11] and affective distress related to an unexpected CD,[12] would result in higher postoperative pain scores and increased opioid intake compared to CD without prior labor (unlabored CD).”*

Comment 5: Line 81: The survey study was performed on women who underwent scheduled and intrapartum CD at six academic medical centers in the United States between September 2014 and March 2016. Participants represented a convenience sample of women who had undergone either elective or unplanned cesarean delivery…

*Response 5: The wording has been adjusted for consistency – “The survey study was performed on women who underwent unlabored and intrapartum CD at six academic medical centers in the United States between September 2014 and March 2016. Participants represented a convenience sample of women who had undergone cesarean delivery…”*

Comment 6: Line 197: Greater opioid consumption after an intrapartum CD, compared with that after scheduled CD, has been previously observed

*Response 6: The wording has been adjusted for consistency – “Greater opioid consumption after an intrapartum CD, compared with that after unlabored CD, has been previously observed.”*

Comment 7: Line 255: In conclusion, women with an unplanned intrapartum CD have modestly higher opioid consumption in the first 2 weeks after delivery.

*Response 7: The wording has been adjusted for consistency – “In conclusion, women with an intrapartum CD have modestly higher opioid consumption in the first two weeks after delivery.”*

**Reviewer 4**

Comment 1: The manuscript seems to be well-written and previous review comments are sound and the authors responded accordingly. I have a comment about the exclusion criteria. Isn't it better to exclude patients with endometriosis, uterine fibroid, and/or adenomyosis? The might influence on the postpartum pain.

*Response 1: While each of these comorbidities may influence postpartum pain and opioid consumption, unfortunately we did not collect data on or account for these conditions. Future work should likely take these conditions into account in verifying our study’s results.*

Comment 2: I would like to know about excluding young patients less than 18. Aged patients might also be probable confounder.

*Response 2: Patients under the age of 18 were excluded due to constraints of subject consent of minors. While there was a statistically significant difference in maternal age between the two groups (Table 1), age was included as a possible confounder in the multivariate analysis, and so differences in age should not be the cause of our reported outcomes.*
